# Tranexamic acid for postpartum bleeding: a systematic review and individual patient data meta-analysis of randomised controlled trials

**DOI:** 10.1016/S0140-6736(24)02102-0

**Published:** 2024-10-26

**Authors:** Katharine Ker, Loïc Sentilhes, Haleema Shakur-Still, Hugo Madar, Catherine Deneux-Tharaux, George Saade, Luis D Pacheco, François-Xavier Ageron, Raoul Mansukhani, Eni Balogun, Amy Brenner, Danielle Prowse, Monica Arribas, Homa Ahmadzia, Rizwana Chaudhri, Oladapo Olayemi, Ian Roberts

**Affiliations:** Clinical Trials Unit, London, School of Hygiene & Tropical, Medicine, London, UK; Department of Obstetrics and Gynecology, Bordeaux University Hospital, Bordeaux, France; Clinical Trials Unit, London, School of Hygiene & Tropical, Medicine, London, UK; Department of Obstetrics and Gynecology, Bordeaux University Hospital, Bordeaux, France; Obstetrical, Perinatal, and Pediatric Epidemiology Research Team, Centre for Research in Epidemiology and Statistics, U1153, INSERM, INRAE, Université Paris Cité, Paris, France; Department of Obstetrics and Gynecology, Eastern Virginia Medical School, Norfolk, VI, USA; Division of Maternal-Fetal Medicine, The University of Texas Medical Branch at Galveston, Galveston, TX, USA; Department of Emergency Medicine, Lausanne University Hospital, University of Lausanne, Lausanne, Switzerland; Clinical Trials Unit, London, School of Hygiene & Tropical, Medicine, London, UK; Clinical Trials Unit, London, School of Hygiene & Tropical, Medicine, London, UK; Clinical Trials Unit, London, School of Hygiene & Tropical, Medicine, London, UK; Clinical Trials Unit, London, School of Hygiene & Tropical, Medicine, London, UK; Clinical Trials Unit, London, School of Hygiene & Tropical, Medicine, London, UK; Inova Health System, Falls Church, VA, USA; George Washington University, Washington, DC, USA; Global Institute of Human Development, Shifa Tameer-e-Millat University, Islamabad, Pakistan; Department of Obstetrics and Gynaecology, University of Ibadan College of Medicine Ibadan, Nigeria; Clinical Trials Unit, London, School of Hygiene & Tropical, Medicine, London, UK

## Abstract

**Background:**

Tranexamic acid is a recommended treatment for women with a clinical diagnosis of postpartum haemorrhage, but whether it can prevent bleeding is unclear. We conducted a systematic review and individual patient data (IPD) meta-analysis of randomised controlled trials to assess the effects of tranexamic acid in women giving birth.

**Methods:**

In this systematic review and IPD meta-analysis, we searched the WHO International Clinical Trials Registry Platform from database inception to Aug 4, 2024 for randomised trials that assessed the effects of tranexamic acid in women giving birth. Trials were eligible if they were prospectively registered, placebo-controlled, included more than 500 women, and had a low risk of bias for random sequence generation and allocation concealment. IPD were requested from the trial investigators. The primary outcomes were the numbers of women with life-threatening bleeding and thromboembolic events. We used a one-stage model to analyse the data and explored whether the effect of tranexamic acid varied by the underlying risk of life-threatening bleeding, type of birth, presence of moderate or severe anaemia, or timing of administration (before or after a diagnosis of postpartum haemorrhage). This study is registered with PROSPERO, CRD42022345775.

**Findings:**

We analysed data on 54 404 women from five trials. We obtained IPD for 43 409 women from four trials and aggregate data on 10 995 women from one trial. All trials had a low risk of bias. Life-threatening bleeding occurred in 178 (0·65%) of 27 300 women in the tranexamic acid group versus 230 (0·85%) of 27 093 women in the placebo group (pooled odds ratio [OR] 0·77 [95% CI 0·63–0·93]; p=0·008). There was no evidence that the effect of tranexamic acid varied by the underlying risk of life-threatening bleeding, type of birth, presence of moderate or severe anaemia or timing of administration. No significant difference was identified between tranexamic acid and placebo groups with regard to thromboembolic events: 50 (0·2%) of 26 571 women in the tranexamic acid group had fatal or non-fatal thromboembolic events versus 52 (0·2%) of 26 373 women in the placebo group (pooled OR 0·96 [0·65–1·41]; p=0·82) with no significant heterogeneity identified in the subgroup analyses.

**Interpretation:**

Tranexamic acid reduces the risk of life-threatening postpartum bleeding. We found no evidence that tranexamic acid increases the risk of thrombosis. Although we do not recommend the use of tranexamic acid in all women giving birth, consideration should be given to its use before a diagnosis of postpartum haemorrhage in women at high risk of death.

**Funding:**

The Bill & Melinda Gates Foundation.

## Introduction

Tranexamic acid reduces mortality in women with a clinical diagnosis of postpartum haemorrhage. In the randomised, placebo-controlled, WOMAN trial,^1^ when given within 3 h of birth, tranexamic acid decreased bleeding deaths by about a third.^1^ In this trial, no discernible increase in thromboembolic events were observed with tranexamic acid, although the effect estimates were imprecise.^1^ WHO recommends tranexamic acid for all women with a clinical diagnosis of postpartum haemorrhage.^2^

Administration of tranexamic acid before a diagnosis of postpartum haemorrhage is made could further reduce maternal mortality. Many women die soon after the onset of bleeding and tranexamic acid is more effective when given early.^3^ However, this would mean treating a much larger number of women. Although all women bleed to some extent after birth, in most women the bleeding is well tolerated. Administration of tranexamic acid to all women before a diagnosis of postpartum haemorrhage is made would expose many women to the potential adverse effects of tranexamic acid, such as an increase in thrombosis, but only a small proportion of women would benefit.

To assess the balance of benefits and harms of tranexamic acid, precise estimates of the effects of tranexamic acid are needed in addition to an understanding of whether they vary with the underlying risks of bleeding and thrombosis. We conducted a systematic review and individual patient data (IPD) meta-analysis using data from large, high-quality randomised trials to quantify the effects of tranexamic acid in women giving birth.

## Methods

### Search strategy and selection criteria

This systematic review with IPD meta-analysis was prospectively registered on PROSPERO (CRD42022345775), and the protocol has been published previously.^4^ We report this systematic review and IPD meta-analysis in accordance with the PRISMA-IPD statement.^5^ Briefly, one author (KK) searched the WHO International Clinical Trials Registry Platform (appendix p 2) from database inception to Aug 4, 2024, for prospectively registered, randomised, placebo-controlled trials assessing the effectiveness of tranexamic acid in women giving birth. Full search terms are in the appendix (p 2). Eligible trials were prospectively registered, randomised, placebo-controlled, included at least 500 women, and had a low risk of bias for random sequence generation and allocation concealment.

### Data collection and risk of bias assessment

We contacted the investigators for each eligible trial to invite them to join our collaboration and requested anonymised IPD for the trial. Data sharing agreements were in place before the transfer of data. Data were securely transferred and stored at the London School of Hygiene & Tropical Medicine Clinical Trials Unit (London, UK) on a server with access restricted to authorised personnel. On receipt, IPD were checked for consistency and completeness by one author (KK), with any queries referred back to the relevant trialist. Two authors (EB and KK) extracted aggregate data from the trial reports using a form designed using Excel specifically for the purpose of this study. Aggregate data on the characteristics of the trials, the methods, participants, interventions and outcomes were extracted. Aggregate data on the prespecified outcomes were also extracted for cross-checking purposes against the individual patient datasets and for use in the analysis if IPD were not available. The same authors (EB and KK) assessed the risk of bias using a modified version of Cochrane’s risk of bias tool.^6^ Any disagreements between the two authors in the extracted data or risk of bias assessments were resolved through discussion.

Institutional review board approval for this study was not required because this study involved the analysis of existing trial data. Each included trial had previously received all the appropriate ethical approvals. The current study did not require further recruitment or data collection from patients, and the analysis did not include identifiable data.

### Outcomes

The primary effectiveness outcome was life-threatening bleeding (a composite outcome defined as death or any of the following surgical interventions for bleeding within 24 h of birth: laparotomy, embolisation, uterine compression sutures, or arterial ligation). The primary safety outcome was fatal or non-fatal thromboembolic events (myocardial infarction, stroke, deep vein thrombosis, or pulmonary embolism) up to the end of follow-up for each trial. The main secondary effectiveness outcome was clinically significant bleeding (a composite outcome defined as life-threatening bleeding or the receipt of an intervention for bleeding within 24 h). Other efficacy outcomes were death within 24 h; death due to bleeding; a shock index (heart rate divided by systolic blood pressure) of 1·4 or greater; surgical intervention for bleeding within 24 h; hysterectomy for bleeding within 24 h (stratified by timing of tranexamic acid); blood transfusion; peripartum change in haemoglobin concentration; transfer to higher level of care; and administration of additional uterotonics. For the shock index, we used a higher threshold (≥1·4) than is typically used in clinical practice as an indicator of haemodynamic instability, to increase the specificity of this outcome and to reduce bias. Secondary safety outcomes were myocardial infarction; stroke; deep vein thrombosis; pulmonary embolism; sepsis; and death or thrombotic events in the neonate. We also assessed maternal breastfeeding and maternal quality of life and did post-hoc analyses to estimate the effects of tranexamic acid on seizures, vomiting, dizziness, and photopsia.

The focus of this review on life-threatening bleeding is consistent with WHO efforts to reduce maternal deaths as described in the WHO postpartum haemorrhage Roadmap.^7^ Although widely used in trials of tranexamic acid for postpartum bleeding, we opted to not include endpoints based on measures of blood loss as outcomes in this review. Postpartum blood loss is difficult to measure accurately, and the evidence underpinning endpoints based on bleeding thresholds such as 500 mL blood loss or higher is unclear.^8^ Furthermore, the clinical relevance of such outcomes and their importance to women is uncertain.

### Data analysis

We used a one-stage model to analyse the data. We used a logistic regression model (STATA logistic command) with a fixed term to account for variation in event rate by trial. If IPD were not available, we extracted aggregate data from trial reports and incorporated them into the analyses using the reconstructed IPD approach.^9^ For binary outcomes, we calculated pooled odd ratios (ORs) with 95% CIs and for continuous outcomes we calculated the pooled mean difference and 95% CI. To assess for heterogeneity in the treatment effect by trial, we included an interaction term for the trial indicator and treatment effect variables in the model. We used a likelihood ratio test to check if the interaction term improved model fit. We considered a p value of less than 0·05 to indicate heterogeneity between trials.

We did subgroup analyses to explore whether the effects of tranexamic acid on life-threatening bleeding, clinically significant bleeding, and thromboembolic events varied by type of birth (vaginal *vs* caesarean), maternal anaemia at baseline (none or mild [haemoglobin ≥100 g/L] *vs* moderate or severe [haemoglobin ≤99 g/L), and timing of tranexamic acid administration (before diagnosis of postpartum haemorrhage *vs* after diagnosis of postpartum haemorrhage). We also explored whether the effects of tranexamic acid on bleeding and thrombosis varied by the underlying risk of these outcomes. For this, we developed prognostic models for the outcomes of life-threatening bleeding, fatal and non-fatal thromboembolic events, and clinically significant bleeding using the IPD from the included trials. Subgroup analyses were carried out by including a subgroup variable in the model. We used separate models for each subgroup analysis rather than including all subgroup variables in one model. We also explored whether the effects of tranexamic acid on bleeding and thrombosis varied by the underlying risk of these outcomes. To assess heterogeneity between subgroups, we included an interaction term for the subgroup variable and the trial indicator variable in our model. We used a likelihood ratio test to check if this interaction term improved model fit. For all subgroups, unless there was strong evidence against homogeneity of effects between subgroups (p<0·001), we considered the pooled effect estimates to be the most reliable guide to the approximate effects in all women.

We used sensitivity analyses to explore the robustness of the results to the inclusion of aggregate data from trials for which IPD were not available. Specifically, the analyses were repeated excluding the aggregate data and the results compared with those from the primary analyses.

Statistical analyses were done using STATA (version 18.0). We used the Grading of Recommendations, Assessment, Development, and Evaluation approach to rate the quality of the evidence for life-threatening bleeding, fatal or non-fatal thromboembolic events, clinically significant bleeding, death within 24 h, shock index of 1·4 or greater, surgical interventions for bleeding within 24 h, and peripartum change in haemoglobin concentration.

### Role of the funding source

The funder had no role in the study design, in the collection, analysis, or interpretation of data, or writing of the report.

## Results

**Figure 1:**
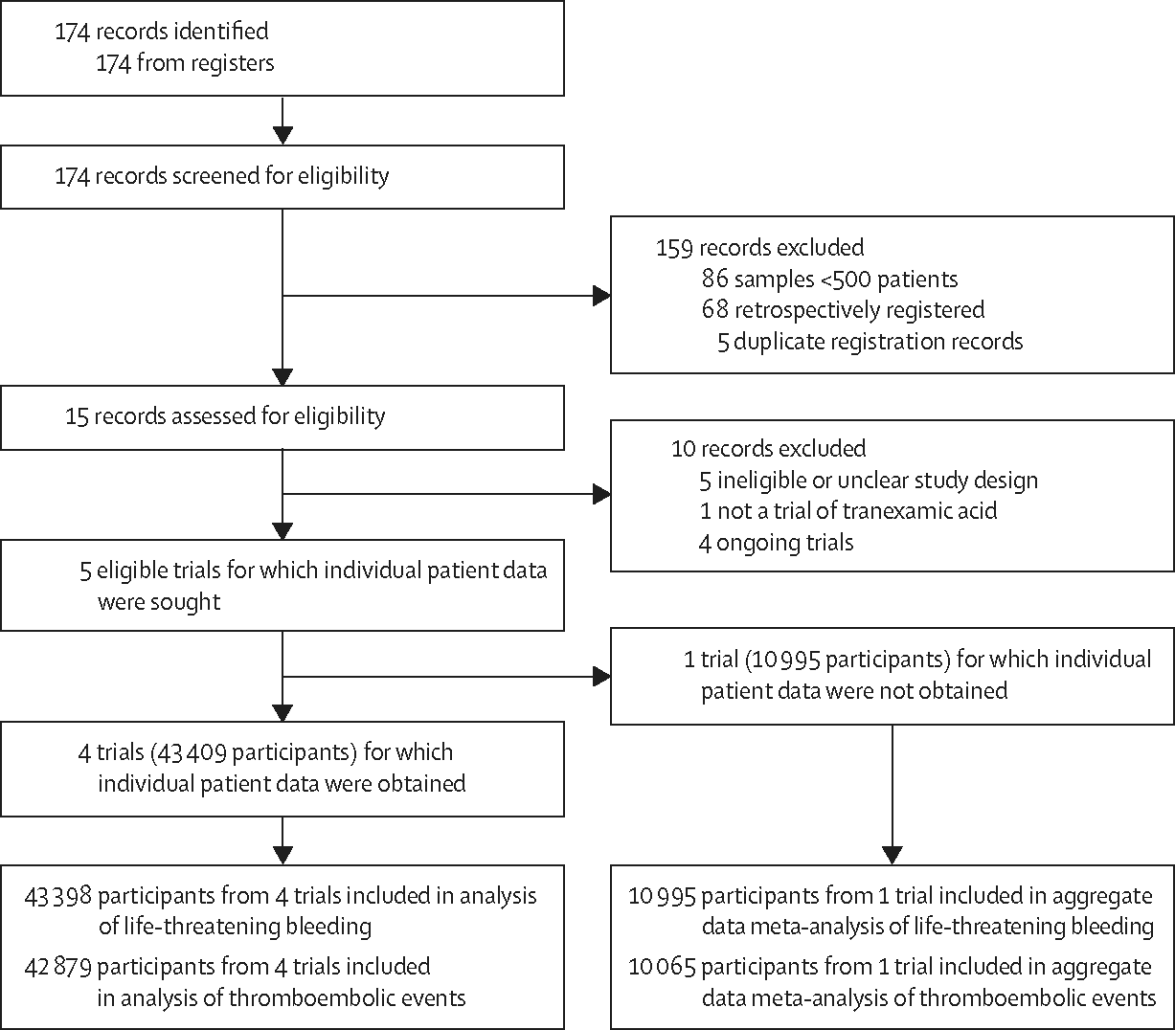
Study selection

Our search identified 174 registration records, of which 86 records referred to trials involving fewer than 500 patients, 68 were retrospectively registered, and five were duplicates. Of the remaining 15 records for which the full registration records or protocols were obtained, a further 10 trials were excluded because they were ongoing, had an unclear or ineligible trial design, or did not assess tranexamic acid. Five completed trials^1,10–13^ met the inclusion criteria, three of which had been completed before publication of our study protocol. Although the trial results were not published at the time we ran our searches, the WOMAN-2 trialists shared IPD for inclusion in this analysis. We obtained IPD for a total of 43 409 women from four included trials (figure 1). Although trialists involved in the TXA Maternal Fetal Medicine Units network (TXA-MFMU) trial agreed to participate, the anonymised individual patient dataset had not been prepared in time to enable inclusion in our analysis; we therefore extracted aggregate data on 10 995 women for this trial from the published trial report (figure 1). A list of the excluded trials with the reasons for their exclusion is shown in the appendix (p 3). Key baseline characteristics of the included trials are shown in table 1 (appendix pp 4–5).

The included trials randomly assigned 54 758 women and provided outcome data for 54 404 women (99·4%). Of the 54 404 women for whom outcome data were available, 33 148 (60·9%) gave birth vaginally, 21 251 (39·1%) gave birth by caesarean, and the type of birth was unknown for five (<0·1%) women. The type of birth was unknown for five women in the WOMAN^1^ trial. The WOMAN^1^ and WOMAN-2^10^ trials recruited mostly in middle-income countries (primarily Nigeria, Pakistan, Tanzania, and Kenya) and the TRAAP,^11^ TRAAP-2,^12^ and TXA-MFMU^13^ trials recruited women in high-income countries (France and USA).

The mean maternal age and baseline haemoglobin concentrations differed by trial (table 1). Gestational age was similar across trials, but mean birthweight was about 500 g lower in the WOMAN-2 trial than in the TRAAP trials. No stillbirths were recorded in the TRAAP trials, whereas stillbirth affected nearly one in ten of the women included in the WOMAN trials.

The route and dose of tranexamic acid used was similar in all five trials with four trials^10–13^ assessing a single dose of 1 g intravenous tranexamic acid or placebo. In the WOMAN trial, 5747 (28·7%) of 20 021 women received a second 1 g dose if the bleeding continued or restarted within 24 h, as per the trial protocol. In the WOMAN trial,^1^ all women were randomly assigned after the diagnosis of postpartum haemorrhage, but in the other four trials women were randomly assigned after birth but before the diagnosis of postpartum haemorrhage.

The included trials had a low risk of bias for all domains (appendix p 6). As per the inclusion criteria, all trials were prospectively registered, placebo-controlled, and used a secure method of allocation concealment. Participants, caregivers, and outcome assessors were unaware of allocation status in all trials and there were minimal missing data. We had no concerns regarding data completeness or integrity.

As prespecified in the study protocol, we ran sensitivity analyses to explore the robustness of our results to the use of aggregate data when IPD were not available. Because similar results were obtained with and without inclusion of the aggregate data, we report results that include aggregate data wherever possible.

The available data did not permit the development of prognostic models with sufficient accuracy for thromboembolic events and clinically significant bleeding for use in these analyses; there were too few events to develop a model for thromboembolic events and the discrimination of the model for the clinically significant bleeding outcome was judged to be too inaccurate. Thus, we only conducted the subgroup analysis by underlying risk for the life-threatening bleeding outcome, for which we used the prognostic model to assign data for each woman to a category based on the distribution of predicted risks from the model. Women with a risk greater than or equal to the median (≥2 cases per 1000 women) were considered at high risk and those with a risk less than the median (<2 cases per 1000 women) were considered at low risk (appendix pp 7–9).

**Figure 2:**
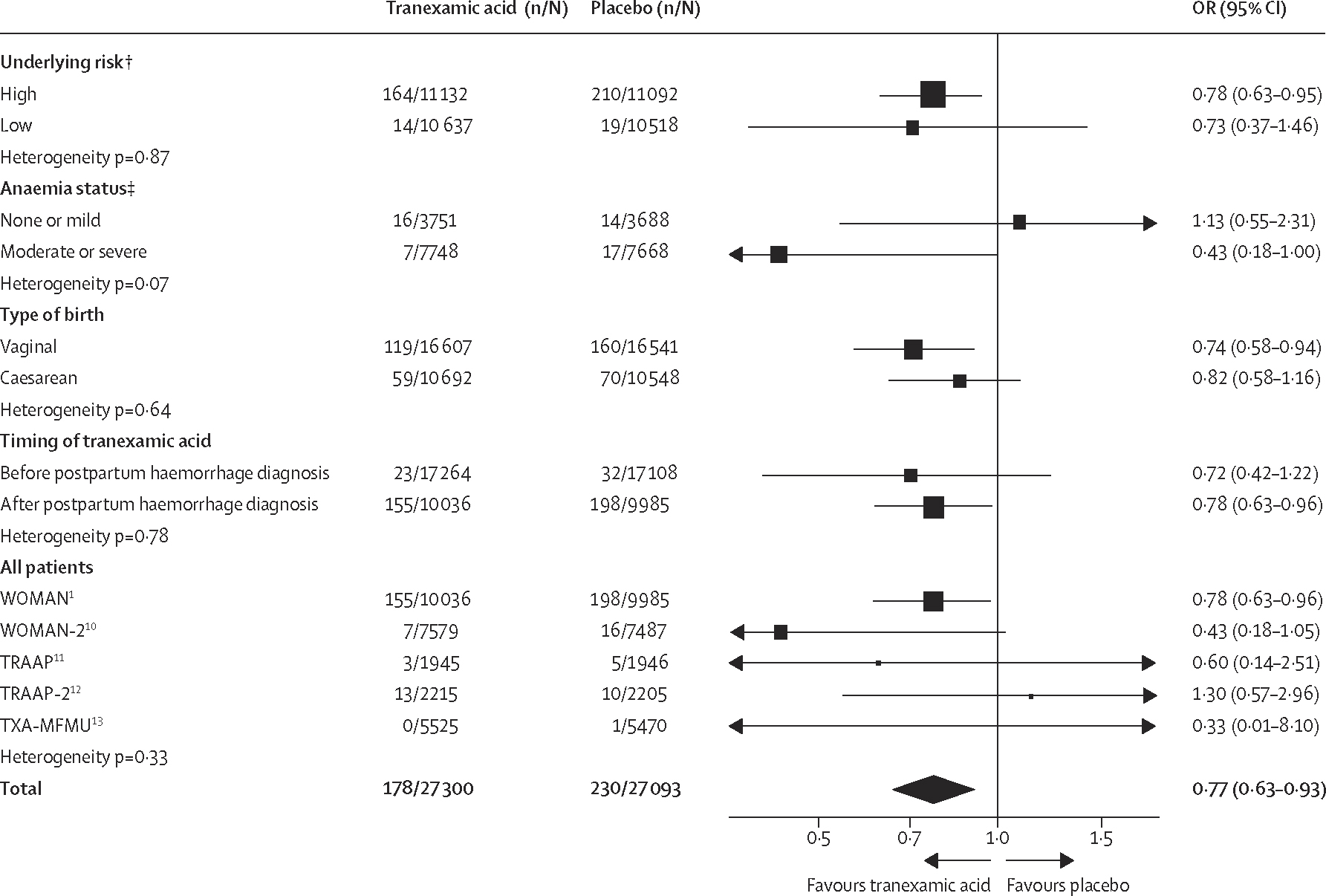
Effect of tranexamic acid on life-threatening bleeding* OR=odds ratio. *Defined as death or ≥1 of the following surgical interventions for bleeding within 24 h after giving birth; laparotomy, embolisation, uterine compression sutures, or arterial ligation. Due to unavailability of individual patient data on surgical procedures within 24 h from the WOMAN and TXA-MFMU trials, only deaths within 24 h were included in this outcome for these trials. †Includes data from the WOMAN, WOMAN-2, TRAAP, and TRAAP-2 trials as individual patient data were not available from the TXA-MFMU trial to facilitate stratification by underlying risk. ‡Includes data from the WOMAN-2, TRAAP, and TRAAP-2 trials as individual patient data on anaemia status at baseline were not available from the WOMAN and TXA-MFMU trials.

We included data from five trials including 54 393 women for the primary outcome of life-threatening bleeding (figure 2). Life-threatening bleeding occurred in 178 (0·65%) of 27 300 women in the tranexamic acid group versus 230 (0·85%) of 27 093 women in the placebo group (pooled OR 0·77 [95% CI 0·63–0·93]; high-quality evidence). No heterogeneity was identified between trials (heterogeneity p=0·33). There was no evidence that the effectiveness varied by the underlying risk of life-threatening bleeding (heterogeneity p=0·87), type of birth (heterogeneity p=0·64), presence or absence of moderate or severe anaemia (heterogeneity p=0·07), or whether or not women were randomly assigned before or after the diagnosis of postpartum haemorrhage (heterogeneity p=0·78).

**Figure 3:**
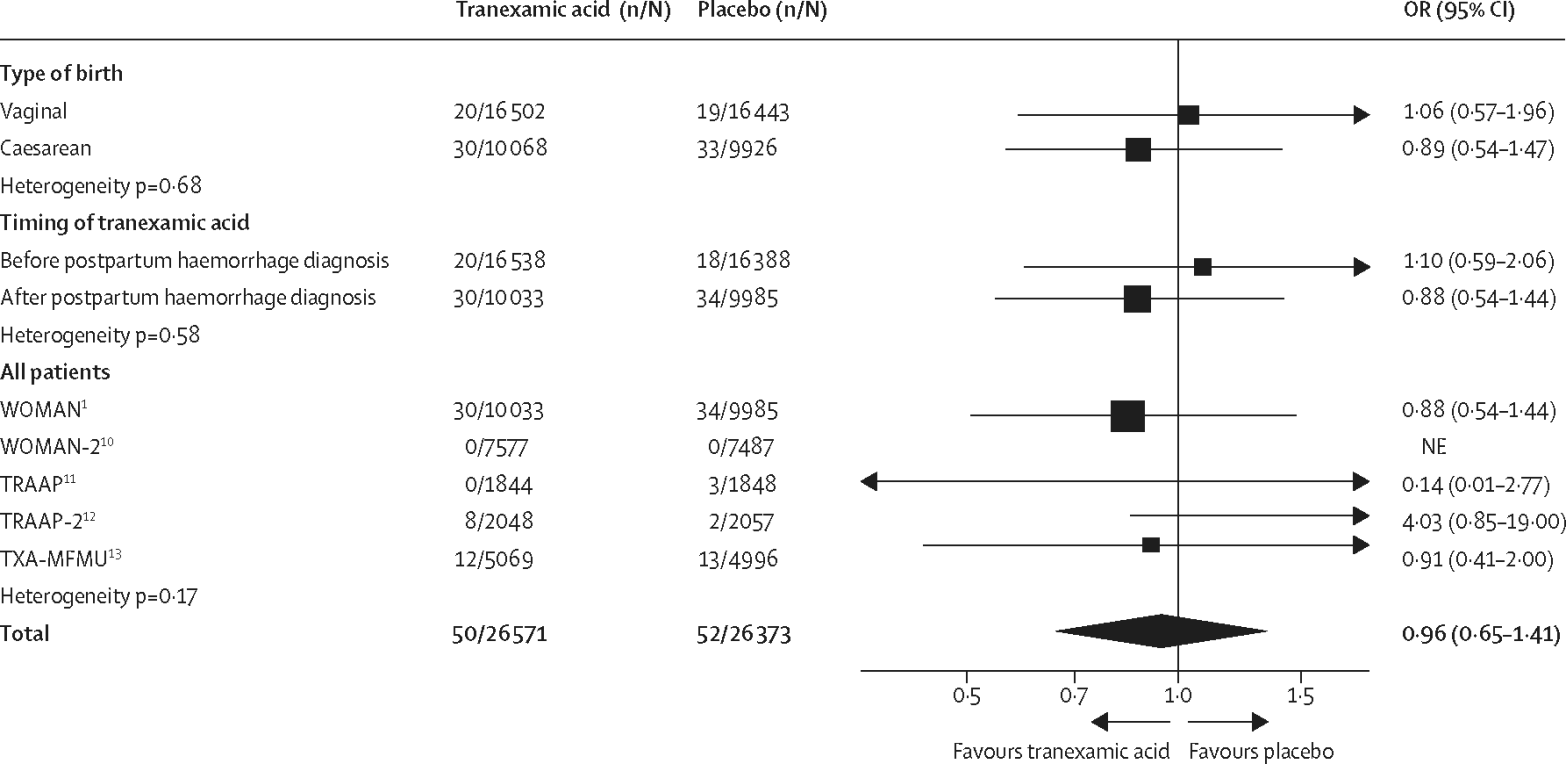
Effect of tranexamic acid on fatal and non-fatal thromboembolic events* OR=odds ratio. NE=not estimable. *Myocardial infarction, stroke, deep vein thrombosis, or pulmonary embolism up to the end of follow-up for each trial. In both WOMAN trials, thromboembolic events were measured at death, hospital discharge, or on day 42 if women remained in hospital; in both TRAAP trials at 3 months after giving birth; and in the TXA-MFMU trial at 6 weeks after giving birth.

We included data from five trials on 52 944 women for the primary safety outcome of fatal or non-fatal thromboembolic events (figure 3). No significant differences were identified between groups with regard to the odds of thrombosis: 50 (0·2%) of 26 571 women in the tranexamic acid group had fatal or non-fatal thromboembolic events versus 52 (0·2%) of 26 373 women in the placebo group (pooled OR 0·96 [95% CI 0·65–1·41]; low-quality evidence). No heterogeneity was identified between trials (heterogeneity p=0·17). There was no evidence that the effect varied by type of birth (heterogeneity p=0·68), or whether or not women were randomly assigned before or after the diagnosis of postpartum haemorrhage (heterogeneity p=0·58). Due to an absence of thromboembolic events in women with moderate or severe anaemia, we could not explore the effect of anaemia at baseline for this outcome.

Three of the four trials^10–12^ that randomly assigned women before the diagnosis of postpartum haemorrhage contributed IPD on 23 370 women for the clinically significant bleeding outcome (table 2). No significant difference in the odds of clinically significant bleeding was identified between groups (1577 [13%] of 11 734 women in the tranexamic acid group *vs* 1600 [14%] of 11 636 women in the placebo group; pooled OR 0·97 [95% CI 0·90–1·05]; high-quality evidence). There was no heterogeneity between trials (heterogeneity p=0·11). There was no heterogeneity by type of birth (heterogeneity p=0·39) or the presence or absence of moderate or severe anaemia (heterogeneity p=0·06).

Analysis of other secondary outcomes combined data (where available) from all trials irrespective of whether or not women had a diagnosis of postpartum haemorrhage at baseline, unless otherwise indicated.

Compared with women in the placebo group, there were fewer deaths within 24 h (pooled OR 0·76 [95% CI 0·62–0·94]; high-quality evidence) and fewer deaths from bleeding (0·81 [0·66–1·00]) among women in the tranexamic acid group. No significant difference was identified between groups with regard to the proportion of women with a shock index of 1·4 or greater (0·97 [0·76–1·24]; high-quality evidence).

No differences were identified between tranexamic acid and placebo groups with regard to surgical interventions (pooled OR 0·79 [95% CI 0·43–1·44]; moderate-quality evidence), hysterectomy when given before diagnosis of postpartum haemorrhage (1·08 [0·48–2·45]), or after diagnosis of postpartum haemorrhage (1·02 [0·87–1·20]), blood transfusion (1·00 [0·95–1·04]) or transfer to a higher level of care (admission to an intensive care unit in all cases; 1·00 [0·91–1·10]). Fewer women received additional uterotonics in the tranexamic acid group than the placebo group (0·92 [0·86–0·98]), but there was statistical heterogeneity between trials (heterogeneity p=0·01). The mean peripartum decrease in haemoglobin concentration was smaller in the tranexamic acid group than the placebo group (pooled mean difference 0·64 g/L [95% CI 0·39–0·89]; moderate-quality evidence), but there was significant heterogeneity between trials (heterogeneity p=0·0002).

No significant differences were identified between groups with regard to the odds of thrombo embolic events (pooled OR 1·33 [95% CI 0·30–5·92] for myocardial infarction; 1·66 [0·60–4·56] for stroke; 0·92 [0·40–2·08] for deep vein thrombosis; 0·81 [0·42–1·53] for pulmonary embolism) or sepsis (1·01 [0·83–1·23]). In post-hoc analysis, no differences were identified between groups with regard to the odds of seizures (0·97 [0·65–1·46]).

There was no evidence for any effect of tranexamic acid on breastfeeding or neonatal outcomes in breastfed babies. We were unable to assess the effect of tranexamic acid on the prespecified secondary outcome of death or thromboembolic events in neonates exposed to tranexamic acid via placental transfer, because tranexamic acid was not administered before cord clamping in any of the included trials

When we compared the results from the one-stage method with those obtained from the two-stage method, the results were similar.

Maternal satisfaction and quality of life were assessed in the TRAAP, TRAAP-2, WOMAN, and WOMAN-2 trials; however, the methods used, and timing of the measurements were too heterogeneous for a pooled analysis. Results of the individual trials showed no evidence of a difference in depression, anxiety, or fatigue between the two groups (appendix p 10).

Results of the post-hoc analyses of the effect of tranexamic acid on other less severe side-effects (vomiting, dizziness, and photopsia), suggest that tranexamic acid might increase the odds of vomiting, however, significant heterogeneity was identified between trials. There was no evidence that tranexamic acid increased dizziness or photopsia (appendix p 11).

Because we included only prospectively registered, large, randomised trials, the risk of small study effects is lower than in systematic reviews that include small trials. We did not examine funnel plots or conduct tests for funnel plots asymmetry because these methods should only be used when there are ten or more trials included in the meta-analysis.^14^

## Discussion

Our results suggest that tranexamic acid reduces the risk of life-threatening bleeding after childbirth. There was no evidence that the odds ratio varies with underlying risk of life-threatening bleeding, the presence or absence of moderate or severe anaemia, the type of birth, or whether or not a diagnosis of postpartum haemorrhage has been made. Because thromboembolic events were rare in the included trials the effect estimate is imprecise, and we cannot exclude a modest increase in thrombosis with tranexamic acid.

To reduce bias, we excluded trials with fewer than 500 participants. A previous systematic review of the available trials showed that including the smaller clinical trials in this area might not improve the validity of the results.^15^ Furthermore, small trials may be underpowered to assess effects on life-threatening bleeding, and there is a greater risk of selective reporting and small study effects when such trials are included. All of the included trials were at low risk of bias; they were prospectively registered, had well concealed allocation, were double-blind, and had minimal missing data. These trials should provide the most reliable estimates of the effects of tranexamic acid. We obtained individual patient-level data from four of the five trials that met our inclusion criteria, with data on 43 409 women. We used aggregate data from one trial with data available for 10 995 women. Our sensitivity analyses showed that the results were similar with and without the inclusion of the aggregate data.

Although the included trials share many similarities, there are also differences, particularly between the patients’ characteristics, which might be relevant when interpreting our results. There were differences in the prevalence of risk factors such as stillbirths, pre-existing hypertensive diseases, placental abnormalities, assisted vaginal births, and the use of epidural analgesia in vaginal births. These factors often varied considerably between trials. Whether such differences would have an effect on the relative treatment effects of tranexamic acid is unclear.

As prespecified in our protocol, we did not include hysterectomy among the surgical interventions in our composite outcome for life-threatening bleeding. Previous trials in women with postpartum haemorrhage found that the decision to do a hysterectomy was often made before randomisation or afterwards for reasons unrelated to the severity of bleeding, such as hysterectomy to remove an adherent placenta.^16^ This should not be the case for other surgical procedures that are specific interventions to control severe bleeding. Including outcome events that could not have been affected by the trial treatment would bias the effect estimates towards the null.^17^ This bias would be less likely in trials of tranexamic acid before the diagnosis of postpartum haemorrhage and so we might have been overcautious. Nevertheless, when we repeated our analysis including the hysterectomy data from these trials, the overall result was similar (data not shown).

Although there were fewer deaths among women administered tranexamic acid treatment than placebo, no significant differences were identified between groups with regard to the number of women receiving a blood transfusion or the number of women transferred to a higher level of care. Randomised trials of tranexamic acid in patients with trauma also found fewer bleeding deaths but no reduction in blood transfusion.^18^ In many low-income and middle-income countries, blood is a scarce resource and the receipt of a blood transfusion depends as much on the availability of blood as the patient’s condition.^19^ Similar concerns apply when deciding whether to transfer a patient to a higher level care. Furthermore, in women giving birth, transfusion is often given for pre-existing anaemia. Blood transfusion might not be sufficiently specific for use as an outcome measure in acute haemorrhage trials.^20^

We found strong evidence that tranexamic acid reduces life-threatening bleeding. This result was mainly driven by data from the WOMAN trial,^1^ which unlike the other included trials, involved women with a diagnosis of postpartum haemorrhage at baseline. We found no statistical evidence that the treatment benefit varied between trials or between subgroups. We found no evidence that tranexamic acid reduces our composite endpoint of clinically significant bleeding, although tranexamic acid was associated with decreased use of additional uterotonics and a smaller reduction in peripartum haemoglobin concentrations, suggestive of reduced bleeding. By defining our outcome of clinically significant bleeding according to whether a woman received one or more interventions for bleeding, we sought to overcome the limitations of an outcome based on estimated blood loss. However, it is possible that the decision to give some of the treatments that comprised our composite endpoint depended more on local clinical practice than on the extent of bleeding, and this might have introduced bias.

It is possible that in the included trials, tranexamic acid was administered too late to reduce the risk of clinically significant bleeding. Although there is no evidence of a harmful effect to the neonate, all included trials administered tranexamic acid after the umbilical cord was cut or clamped to avoid the transplacental passage of the medication. In most trials, tranexamic acid was given by slow intravenous infusion, which would introduce a further treatment delay. However, in some cases severe bleeding might have already started. In the WOMAN-2 trial,^10^ bleeding from birth canal trauma or from the placental bed could have started many minutes before the umbilical cord was clamped. Since uterine blood flow at term can be as high as 800 mL per min, severe bleeding can occur rapidly.^21^ In these women, giving tranexamic acid after cord clamping might be too late to prevent postpartum haemorrhage, but not too late to prevent life-threatening bleeding. Randomised trials in surgery show that tranexamic acid given before incision substantially reduces bleeding and improves patient outcomes,^22,23^ suggesting that early administration could similarly benefit women during caesarean birth. The ongoing I’M WOMAN trial (NCT05562609) with a planned sample of 30 000 women will provide valuable evidence on the effects of giving tranexamic acid before vaginal birth or skin incision in caesarean birth.

The inadvertent intrathecal injection of tranexamic acid can cause serious harms.^22^ There were no such cases in any of the included trials. However, with the increasing use of tranexamic acid to reduce life-threatening bleeding, measures should be taken to avoid this error.

Our results differ from those of previous reviews of aggregate data from trials of tranexamic acid for preventing postpartum bleeding.^24–26^ Systematic reviews that included smaller trials (≤500 participants) found a reduction in postpartum haemorrhage and blood transfusion, although the studies were not powered to make reliable inferences about the effect of tranexamic acid on life-threatening bleeding. Our decision to limit our review to large, prospectively registered, high-quality trials, including the most recent trial evidence, might account for this difference.

In conclusion, tranexamic acid reduces life-threatening bleeding, defined as death or surgical intervention for bleeding within 24 h. There is no evidence that tranexamic acid reduces other non-fatal bleeding endpoints, although fewer women in the tranexamic acid group received additional uterotonic use than did women in the placebo group, and women given tranexamic acid had higher postpartum haemoglobin concentrations than women in the placebo group. No apparent increase in thromboembolic events was observed with tranexamic acid, but because thrombosis is a rare outcome and was possibly under-reported in some of the trials, we cannot exclude a modest increase. Although administration of tranexamic acid before a diagnosis of postpartum haemorrhage should be considered in women at high risk of death from bleeding, the evidence does not support extending the use of tranexamic acid to all women to prevent postpartum bleeding. For women at higher risk of life-threatening bleeding (eg, ≥1 per 100 women giving birth) the number needed to treat to prevent one woman from developing a life-threatening bleed is fewer than 500. However, for women at lower risk (eg, ≤1 per 100 000 women giving birth), hundreds of thousands of women would need to be treated to prevent one woman from developing a life-threatening bleed. In women at low risk of life-threatening bleeding, even a small number of adverse events from inadvertent intrathecal injection of tranexamic acid could outweigh any treatment benefit. Caution must be taken with regard to offering tranexamic acid to women at low risk of life-threatening bleeding since the balance of benefit and potential harm is more uncertain in these women. Further research including cost-effectiveness modelling is needed to inform treatment decisions.

## Supplementary Material

supplementary info

## Figures and Tables

**Table 1:** Characteristics of the included trials

	WOMAN^1^	WOMAN-2^10^	TRAAP^11^	TRAAP-2^12^	TXA-MFMU^13^

**Participants**					
Randomly assigned, n	20 060	15 068	4079	4551	11 000
Included in analysis	20 021	15 066	3891	4431	10 995
Setting	LMICs	LMICs	HIC	HIC	HIC
Age, years (mean [SD])	28·3 (5·7)	27·2 (5·6)	30·3 (4·9)	33·4 (5·3)	30·1 (5·8)
Type of birth	Vaginal and caesarean*	Vaginal	Vaginal	Caesarean	Caesarean
Prebirth haemoglobin, g/L (mean [SD])	Not available	83 (12)	118 (10)	118 (11)	Not available
Diagnosis of postpartum haemorrhage at baseline	Yes	No†	No	No	No
Stillbirths	1986 (9·9%)	1036 (6·9%)	0	0	Not available
Antepartum haemorrhage	Not available	435 (2·9%)	88 (2·3%)	62 (1·4%)	Not available
>1 fetus	1045 (5·2%)	583 (3·9%)	0	319 (7·2%)	458 (4·2%)
Gestational diabetes	Not available	61 (<1%)	420 (10·8%)	946 (21·3%)	Not available
Hypertensive disorders of pregnancy	Not available	1146 (7·6%)	84 (2·2%)	283 (6·4%)	1924 (17·5%)
Placental abnormalities	1878 (9·4%)	469 (3·1%)	0	0	316 (2·9%)
Assisted vaginal birth	Not available	422 (2·8%)	678 (17·4%)	Not applicable	Not applicable
Planned caesarean	Not available	Not applicable	Not applicable	3145 (71·0%)	5461 (49·7%)‡
Episiotomy	Not available	4355 (28·9%)	900 (23·1%)	Not applicable	Not applicable
Perineal tears	Not available	1788 (11·9%)	2218 (57·0%)	Not applicable	Not applicable
Epidural or spinal analgesia	Not available	6 (<1%)	3808 (97·9%)	4382 (98·9%)	Not applicable
Birthweight, g (mean [SD])	Not available	2812 (557)	3395 (422)	3213 (631)	Not available
Gestational age, weeks (mean [SD])	Not available	37 (3)	=35§	=34§	38 (2)
**Intervention**					
Route	Intravenous	Intravenous	Intravenous	Intravenous	Intravenous
Dose, g	1 or 2¶	1	1	1	1
Timing	After postpartum haemorrhage diagnosis	Before postpartum haemorrhage diagnosis (≤15 min after cord clamping)	Before postpartum haemorrhage diagnosis (≤2 min after birth and cord clamping)	Before postpartum haemorrhage diagnosis (≤3 min after birth and cord clamping)	Before postpartum haemorrhage diagnosis (at cord clamping)
Comparator	Normal saline	Normal saline	Normal saline	Normal saline	Normal saline

The number of women included in the analysis were used as the denominators for the calculation of percentages. LMICs=low-income and middle-income countries. HIC=high-income country.

* 14 191 gave birth vaginally, 5825 by caesarean, and type of birth was unknown for five women.

† Seven women with clinical diagnosis of postpartum haemorrhage were randomly assigned as protocol violations.

‡ Randomisation in the TXA-MFMU trial was restricted to allow for a maximum of 50% scheduled caesarean births.

§ Trial eligibility criterion.

¶ A subset of women (5747 [28·7%] of 20 021) in the WOMAN trial received a second 1 g dose if the bleeding continued or restarted within 24 h, as per the trial protocol.

**Table 2:** Effect of tranexamic acid on secondary maternal and neonatal outcomes

	Contributing trials	Tranexamic acid group (n/N)	Placebo group (n/N)	Pooled OR (95% CI)	Heterogeneity p value

**Secondary maternal outcomes**					
Clinically significant postpartum bleeding*	WOMAN-2,^10^ TRAAP,^11^ and TRAAP-2^12^	1577/11 734	1600/11 636	0·97 (0·90–1·05)	0·11
Death within 24 h	WOMAN,^1^ WOMAN-2,^10^ TRAAP,^11^ TRAAP-2,^12^ and TXA-MFMU^13^	159/27 308	206/27 096	0·76 (0·62–0·94)	0·61†
Death due to bleeding	WOMAN,^1^ WOMAN-2,^10^ TRAAP,^11^ TRAAP-2,^12^ and TXA-MFMU^13^	159/27 307	194/27 097	0·81 (0·66–1·00)	0·52
Shock index ≥1·4	WOMAN-2,^10^ TRAAP,^11^ and TRAAP-2^12^	129/11 549	132/11 448	0·97 (0·76–1·24)	0·82
Surgical intervention for bleeding within 24 h	WOMAN-2,^10^ TRAAP,^11^ and TRAAP-2^12^	19/11 739	24/11 638	0·79 (0·43–1·44)	0·18
Hysterectomy within 24 h (when tranexamic acid given before postpartum haemorrhage diagnosis)	WOMAN-2,^10^ TRAAP,^11^ and TRAAP-2^12^	12/11 745	11/11 642	1·08 (0·48–2·45)	0·35
Hysterectomy within 24 h (when tranexamic acid given after postpartum haemorrhage diagnosis)	WOMAN^1^	303/10 036	296/9985	1·02 (0·87–1·20)	NE
Blood transfusion	WOMAN,^1^ WOMAN-2,^10^ TRAAP,^11^ TRAAP-2,^12^ and TXA-MFMU^13^	7685/27 303	7646/27 094	1·00 (0·95–1·04)	0·60
Transfer to higher level of care	WOMAN,^1^ WOMAN-2,^10^ TRAAP,^11^ TRAAP-2,^12^ and TXA-MFMU^13^	915/26 848	908/26 640	1·00 (0·91–1·10)	0·45
Additional uterotonics	WOMAN-2,^10^ TRAAP,^11^ TRAAP-2,^12^ and TXA-MFMU^13^	1990/17 266	2116/17 109	0·92 (0·86–0·98)	0·01
Myocardial infarction	WOMAN,^1^ WOMAN-2,^10^ TRAAP,^11^ TRAAP-2,^12^ and TXA-MFMU^13^	4/27 025	3/26 848	1·33 (0·30–5·92)	0·35
Stroke	WOMAN,^1^ WOMAN-2,^10^ TRAAP,^11^ TRAAP-2,^12^ and TXA-MFMU^13^	10/27 025	6/26 848	1·66 (0·60–4·56)	0·42
Deep vein thrombosis	WOMAN,^1^ WOMAN-2,^10^ TRAAP,^11^ and TRAAP-2^12^	11/21 502	12/21 378	0·92 (0·40–2·08)	0·03
Pulmonary embolism	WOMAN,^1^ WOMAN-2,^10^ TRAAP,^11^ and TRAAP-2^12^	17/21 502	21/21 378	0·81 (0·42–1·53)	0·58
Sepsis	WOMAN,^1^ WOMAN-2,^10^ TRAAP-2,^12^ and TXA-MFMU^13^	205/25 185	202/25 000	1·01 (0·83–1·23)	0·27
Seizures	WOMAN,^1^ WOMAN-2,^10^ TRAAP,^11^ TRAAP-2,^12^ and TXA-MFMU^13^	46/26 570	47/26 371	0·97 (0·65–1·46)	0·16†
**Neonatal outcomes**					
Not breastfed	WOMAN,^1^ WOMAN-2,^10^ TRAAP,^11^ and TRAAP-2^12^	3656/20 597	3560/20 555	1·03 (0·98–1·08)	0·95
Death or thrombotic event in breastfed baby	WOMAN,^1^ WOMAN-2,^10^ TRAAP,^11^ and TRAAP-2^12^	10/17 015	17/16 938	0·60 (0·28–1·28)	0·20

NE=not estimable.

* Composite outcome defined as life-threatening bleeding or receipt of any of the following interventions for bleeding within 24 h: additional uterotonics, non-trial tranexamic acid, perineal or vaginal packing, manual removal of placenta, uterine tamponade, bimanual compression, external aortic compression, non-pneumatic anti-shock garment, or uterine compression sutures.

† Based on the Wald test.

## Data Availability

Anonymised data from the WOMAN trial are available for download from the FreeBIRD data repository (freebird.lshtm.ac.uk). Anonymised data from the WOMAN-2 will also be available from FreeBIRD after publication of the primary and secondary trial analyses. Request for data from the TRAAP trials can be made to the trials’ steering committees via the lead investigators. Aggregate data from the TXA-MFMU trial are available in the published trial report and in the ClinicalTrials.gov record (https://clinicaltrials.gov/study/NCT03364491?tab=results).

## Research in context

### Evidence before this study

Tranexamic acid is recommended for women with postpartum haemorrhage. However, whether it should also be given to women to prevent bleeding is unclear. Our searches of the literature (MEDLINE, EMBASE, CENTRAL, and PROSPERO databases using subject headings and terms relevant to ‘tranexamic acid’ and ‘postpartum bleeding’) before initiating this study, showed that, although many systematic reviews exist, the results are limited by the inclusion of aggregate data from small and unreliable trials. Reliable estimates of the benefits and harms of tranexamic acid in women giving birth and how these vary by underlying risk and other patient characteristics are needed to inform treatment policy. New evidence from large, high-quality trials, such as the WOMAN-2 trial, provides an opportunity to address this need. This systematic review and individual patient data (IPD) meta-analysis was conducted to set the results from the WOMAN-2 trial in their wider scientific context and to inform WHO policy making on the prevention of maternal death from postpartum haemorrhage (the postpartum haemorrhage Roadmap).

### Added value of this study

We conducted a systematic review and IPD meta-analysis to assess the effect of tranexamic acid on life-threatening bleeding and thrombosis. We used IPD from high-quality trials involving at least 500 women, to investigate how these effects varied by underlying risk of life-threatening bleeding and other patient characteristics. This analysis includes data from 54 404 women from five high-quality trials of tranexamic acid. Tranexamic acid reduced life-threatening bleeding in women giving birth, regardless of the underlying risk, the type of birth, the presence of moderate or severe anaemia, or whether a diagnosis of postpartum haemorrhage had been made. There was no evidence that tranexamic acid increased thrombosis.

### Implications of all the available evidence

Tranexamic acid reduces the risk of life-threatening postpartum bleeding. There is no evidence that tranexamic acid increases the risk of thrombosis. Administration of tranexamic acid to women at high risk of death, before a diagnosis of postpartum haemorrhage is made, warrants consideration in clinical practice.
