# Supplementary material for: Tranexamic acid for postpartum bleeding: a systematic review and individual patient data meta-analysis of randomised controlled trials
Source: Lancet. Author manuscript; Available in PMC 2025 Jun 26. (PMC12197804; doi:10.1016/S0140-6736(24)02102-0)
Supplement: supplementary info [file NIHMS2085243-supplement-supplementary_info.pdf]

THE LANCET

Supplementary appendix

This appendix formed part of the original submission and has been peer reviewed. We post it as supplied by the authors.

Supplement to: Ker K, Sentilhes L, Shakur-Still H, et al. Tranexamic acid for postpartum bleeding: a systematic review and individual patient data meta-analysis of randomised controlled trials. *Lancet* 2024; **404:** 1657–67.

Supplementary appendix

File 1: Search terms for the WHO International Trial Registry Platform

("Tranexamic Acid" OR TXA OR AMCA OR AMCHA OR Amchafibrin OR Anvitoff OR Cyklokapron OR Cyclocapron OR cyklocapron OR Exacyl OR KABI 2161 OR Spotof OR t-AMCHA OR "trans-4-(Aminomethyl)cyclohexanecarboxylic Acid" OR Transamin OR Ugurol OR Lysteda OR Cyclo-F OR Amstat OR Hexacapron OR Hexakapron OR "aminomethylcyclohexanocarboxylic acid" OR amchafibrin OR amikapron OR Amicar OR "Aminocaproic Acid" OR Afibrin OR Amica OR acikaprin OR caprogel OR Capralense OR Capramol OR Caproamin OR Caprocid OR Caprolest OR caprolisine OR CY 116 OR CY-116 OR CY116 OR ekaprol OR Epsamon OR Epsikapron OR Epsicapron OR epsilcapramin OR Hemocaprol OR Hexalense) AND (postpartum OR PPH OR post-partum OR birth OR childbirth OR caesarean OR delivery OR cesarean)

File 2: List of excluded studies

Trial ID	Reason for exclusion
ChiCTR2200062464	Only registration record identified as of October 2023. States recruitment was planned to end by 30/9/23. Open label trial, no blinding of participants or staff. Method of allocation concealment unclear.
Arya 2024	Method of allocation concealment (sealed envelopes) did not meet prespecified inclusion criteria.
EUCTR2018-003960-29-FI	Only registration record identified as of October 2023. Gives planned completion date of end of 2021. No response from investigator for information. Unable to determine if allocation was adequately concealed.
NCT03463993	Open label trial, no blinding of participants or staff. Method of allocation concealment (sealed envelopes) did not meet prespecified inclusion criteria.
NCT04733157	Method of allocation concealment (sealed envelopes) did not meet prespecified inclusion criteria.
NCT03326596	Not RCT (prospective cohort)

File 3: Additional details of the included trials

WOMAN 2017	
**Methods**	International, multicentre, double-blind, randomised controlled trial.
**Setting**	193 hospitals in 21 countries.
**Participants**	20,060 women with a clinical diagnosis of postpartum haemorrhage after a vaginal birth or caesarean section were randomly allocated. 39 randomised women withdrew consent or were lost to follow-up (<0.2%).
**Interventions**	1g intravenous TXA or placebo. If bleeding continued after 30 minutes, or stopped and restarted within 24 hours of the first dose, a second dose of 1g TXA or placebo could be given.
**Outcome data**	IPD for 20,021 women (10,036 in the TXA group and 9985 in the placebo group) were available for inclusion in this analysis.<br>Contributed IPD to the following outcomes of interest to this review; life-threatening bleeding, thromboembolic events, death within 24 hours, death due to bleeding, myocardial infarction, stroke, deep vein thrombosis, pulmonary embolism, hysterectomy for bleeding, blood transfusion, transfer to higher level of care, sepsis, seizures, maternal quality of life, death or thrombotic events in breast-fed babies, breastfeeding.
**Trial information**	Dates of the study: 05/2009 to 04/2017<br>Funding sources: London School of Hygiene & Tropical Medicine, Pfizer, UK Department of Health, Wellcome Trust, and the Bill & Melinda Gates Foundation<br>Trial registration details: NCT00872469 (31/3/2009)<br>REC approval: LSHTM REC #5536 and local approvals specific to each participating country and hospital.

WOMAN-2 2024	
**Methods**	International, multicentre, double-blind, randomised controlled trial.
**Setting**	43 hospitals in Nigeria, Pakistan, Tanzania and Zambia.
**Participants**	15,068 women with moderate or severe anaemia giving birth vaginally were randomly allocated. 2 randomised women were lost to follow-up (0.01%).
**Interventions**	1g TXA or matching placebo (sodium chloride 0.9%) by intravenous injection immediately (within 15 minutes) after the umbilical cord is cut or clamped.
**Outcomes**	IPD for 15,066 women (7579 in the TXA group and 7487 in the placebo group) were available for inclusion in this analysis.<br>Contributed data to the following outcomes of interest to this review; life-threatening bleeding, thromboembolic events, clinically significant postpartum bleeding, death within 24 hours, death due to bleeding, shock index ≥1.4, surgical intervention for bleeding, myocardial infarction, stroke, deep vein thrombosis, pulmonary embolism, hysterectomy for bleeding, peripartum haemoglobin change, additional uterotonics, blood transfusion, transfer to higher level of care, sepsis, seizures, maternal quality of life, death or thrombotic events in breast-fed babies, breastfeeding, vomiting, dizziness.
**Trial information**	Dates of the study: 11/2017 to 9/2023<br>Funding sources: Wellcome Trust & Bill and Melinda Gates Foundation<br>Trial registration details: NCT03475342 (23/3/2018)<br>REC approval: LSHTM REC#15194 and local approvals specific to each participating country and hospital.

TRAAP 2019	
**Methods**	Multicentre, double-blind, randomised controlled trial.
**Setting**	15 maternity units in France.
**Participants**	4079 women 18 years or older, giving birth vaginally to a single live foetus at ≥35 weeks gestation were randomly allocated. 46 women were found to be ineligible or withdrew consent after randomisation and 142 went on to give birth by caesarean and were excluded (4.6%).
**Interventions**	1g intravenous TXA or placebo delivered during the 2 minutes after birth after the routine prophylactic injection of oxytocin at delivery of the anterior shoulder.
**Outcome data**	IPD for 3891 women (1945 in the TXA group and 1946 in the placebo group) were available for inclusion in this analysis.<br>Contributed IPD to the following outcomes of interest to this review; life-threatening bleeding, thromboembolic events, clinically significant postpartum bleeding, death within 24 hours, death due to bleeding, shock index ≥1.4, surgical intervention for bleeding, myocardial infarction, stroke, deep vein thrombosis, pulmonary embolism, hysterectomy for bleeding, peripartum haemoglobin change, additional uterotonics, blood transfusion, transfer to higher level of care, sepsis, seizures, maternal quality of life, death or thrombotic events in breast-fed babies, breastfeeding, vomiting, dizziness, photopsia.
**Trial information**	Dates of the study: 01/2015 to 12/2016<br>Funding sources: French Ministry of Health under the Clinical Research Hospital Program (contract no., PHRCN 1370458 N)<br>Trial registration details: NCT02302456 (17/11/2014)

	REC approval: Ouest II Committee for the Protection of Research Subjects and the French Health Products Safety Agency.

**TRAAP-2 2021**	
**Methods**	Multicentre, double-blind, randomised controlled trial.
**Setting**	27 hospitals in France.
**Participants**	4551 women due to give birth by caesarean were randomly allocated. 112 women were found to be ineligible or withdrew consent after randomisation and 8 went on to give birth vaginally and were excluded (2.5%).
**Interventions**	1g TXA or placebo during the 3 minutes after birth.
**Outcome data**	IPD for 4431 women (2222 in the TXA group and 2209 in the placebo group) were available for inclusion in this analysis.<br>Contributed IPD to the following outcomes of interest to this review; life-threatening bleeding, thromboembolic events, clinically significant postpartum bleeding, death within 24 hours, death due to bleeding, shock index ≥1.4, surgical intervention for bleeding, myocardial infarction, stroke, deep vein thrombosis, pulmonary embolism, hysterectomy for bleeding, peripartum haemoglobin change, additional uterotonics, blood transfusion, transfer to higher level of care, sepsis, seizures, maternal quality of life, death or thrombotic events in breast-fed babies, breastfeeding, vomiting, dizziness, photopsia.
**Trial information**	Dates of the study: 03/2018 to 04/2020<br>Funding sources: French Ministry of Health<br>Trial registration details: NCT03431805 (13/2/2018)<br>REC approval: North-west VI Committee for the Protection of Research Subjects and the French National Agency of Medicine and Health Products Safety.

**TXA-MFMU 2023**	
**Methods**	Multicentre, double-blind, randomised controlled trial.
**Setting**	31 hospitals in the USA.
**Participants**	11,000 women due to give birth by caesarean were randomly allocated. 5 women presented during two different pregnancies; only the first pregnancy was included.
**Interventions**	1g TXA or placebo immediately at cord clamping.
**Outcomes**	Aggregate data for 10,995 women (5525 in the TXA group and 5470 in the placebo group) were available for inclusion in this analysis.<br>Contributed data to the following outcomes of interest to this review; life-threatening bleeding, thromboembolic events, death within 24 hours, myocardial infarction, stroke, pulmonary embolism, hysterectomy for bleeding, peripartum haemoglobin change, additional uterotonics, blood transfusion, transfer to higher level of care, sepsis, seizures, vomiting, dizziness.
**Trial information**	Dates of the study: 03/2018 to 10/2021<br>Funding sources: Eunice Kennedy Shriver National Institute of Child Health and Human Development<br>Trial registration details: NCT03364491 (6/12/2017)<br>REC approval: Approval obtained from the institutional review board at each hospital.

File 4: Risk of bias of included trials

Domain	WOMAN	WOMAN-2	TRAAP	TRAAP-2	TXA-MFMU
**1. Was the allocation sequence adequately generated?**	**Definitely yes (low risk of bias)** Computer-generated randomisation	**Definitely yes (low risk of bias)** Computer-generated randomisation	**Definitely yes (low risk of bias)** Computer-generated randomisation	**Definitely yes (low risk of bias)** Computer-generated randomisation	**Definitely yes (low risk of bias)** Computer-generated randomisation
**2. Was the allocation adequately concealed?**	**Definitely yes (low risk of bias)** Pharmacy-prepared, sequentially numbered drug containers of identical appearance	**Definitely yes (low risk of bias)** Pharmacy-prepared, numbered drug containers of identical appearance	**Definitely yes (low risk of bias)** Pharmacy-prepared, numbered drug containers of identical appearance	**Definitely yes (low risk of bias)** Pharmacy-prepared, numbered drug containers of identical appearance	**Definitely yes (low risk of bias)** Pharmacy-prepared, numbered drug containers of identical appearance
**3. Blinding: Was knowledge of the allocated interventions adequately prevented?**	**Definitely yes (low risk of bias)** Blinding of participants and key study personnel ensured, and unlikely that blinding could have been broken	**Definitely yes (low risk of bias)** Blinding of participants and key study personnel ensured, and unlikely that blinding could have been broken	**Definitely yes (low risk of bias)** Blinding of participants and key study personnel ensured, and unlikely that blinding could have been broken	**Definitely yes (low risk of bias)** Blinding of participants and key study personnel ensured, and unlikely that blinding could have been broken	**Definitely yes (low risk of bias)** Blinding of participants and key study personnel ensured, and unlikely that blinding could have been broken
**4. Was loss to follow-up (missing outcome data) infrequent?**	**Definitely yes (low risk of bias)** Missing outcome data minimal and unlikely to be related to outcome.	**Definitely yes (low risk of bias)** Missing outcome data minimal and unlikely to be related to outcome.	**Definitely yes (low risk of bias)** Missing outcome data minimal and unlikely to be related to outcome.	**Definitely yes (low risk of bias)** Missing outcome data minimal and unlikely to be related to outcome.	**Definitely yes (low risk of bias)** Missing outcome data minimal and unlikely to be related to outcome.
**5. Are reports of the study free of selective outcome reporting?**	**Definitely yes (low risk of bias)** The study protocol is available and all of the study's pre-specified (primary and secondary) outcomes that are of interest in the review have been reported. Furthermore, IPD of all outcomes of interest to this review (if collected) were made available.	**Definitely yes (low risk of bias)** The study protocol is available and all of the study's pre-specified (primary and secondary) outcomes that are of interest in the review have been reported. Furthermore, IPD of all outcomes of interest to this review (if collected) were made available.	**Definitely yes (low risk of bias)** The study protocol is available and all of the study's pre-specified (primary and secondary) outcomes that are of interest in the review have been reported. Furthermore, IPD of all outcomes of interest to this review (if collected) were made available.	**Definitely yes (low risk of bias)** The study protocol is available and all of the study's pre-specified (primary and secondary) outcomes that are of interest in the review have been reported. Furthermore, IPD of all outcomes of interest to this review (if collected) were made available.	**Definitely yes (low risk of bias)** The study protocol is available and all of the study's pre-specified (primary and secondary) outcomes that are of interest in the review have been reported.
**6. Was the study apparently free of other problems that could put it at a risk of bias?**	**Definitely yes (low risk of bias)** No concerns	**Definitely yes (low risk of bias)** No concerns	**Definitely yes (low risk of bias)** No concerns	**Definitely yes (low risk of bias)** No concerns	**Definitely yes (low risk of bias)** No concerns

File 5: Prognostic model for life-threatening bleeding

We used multi-level logistic regression to predict the different outcomes. We included a random effect by trial and by country income level (low, middle or high income country as defined by the World Bank). We removed variables not associated with outcome one by one based on the p-value of the Wald test. We performed a likelihood ratio test after each removal of a variable to test for any difference. We selected all plausible risk factor variables in the model. We tested all plausible interactions between covariates such as age, type of birth, placental abnormalities, antepartum haemorrhage and anaemia. We were careful not to select variables on the causal pathway between covariates and outcome. We assessed collinearity in the model by estimating the variance inflation factor. In addition, we performed sensitivity analysis for variable selection using a lasso model (least absolute shrinkage and selection operator). Continuous variables were included as their linear and polynomial terms, when necessary, after testing for log-linearity of the association. For some continuous variables where linearity was not confirmed, we used splines. As there were few missing values, we performed a complete case analysis.

Equation model for baseline prediction of life-threatening bleeding

Due to collinearity and causal pathway between anaemia, abruption, and antepartum haemorrhage. The model was developed with the variable abruption only, considering that anaemia could be on the causal pathway. Abruption is the leading causes of antepartum haemorrhage.
Mixed logistic regression with random effect on country were performed for each model.
Predicted probability of life-threatening bleeding $= \frac{1}{1+ e^{-S}}$
Where *S* =

Model
S= -0.351632 - 8·138668 + (age_cat * 0·3730297) + (3·084818 if SBP <80 mmHg) or (1·307297 if 100 > SBP ≥ 80) + (3·503377 if abruption) - (0·4830775 if placenta abnormalities) + (0·7806479 if hypertensive disease) + (0·5865626 if primi gravida) – (0·2562096 if TXA treatment)

Age categories
0=<20
1= 20-25
2= 26-29
3=30-39
4=≥40

Performance of the regression models

	Model 1
Brier Score	0·0078
AUROC	0·849 (0·827-0·870)
Calibration in the large	-0·0000269
Calibration slope	0·98 (0·94-1·02)
Ratio E/O	1·00 (0·90-1·10)
Internal-external validation AUROC (cross validation)	0·864 (0·846-0·883)

ROC curve

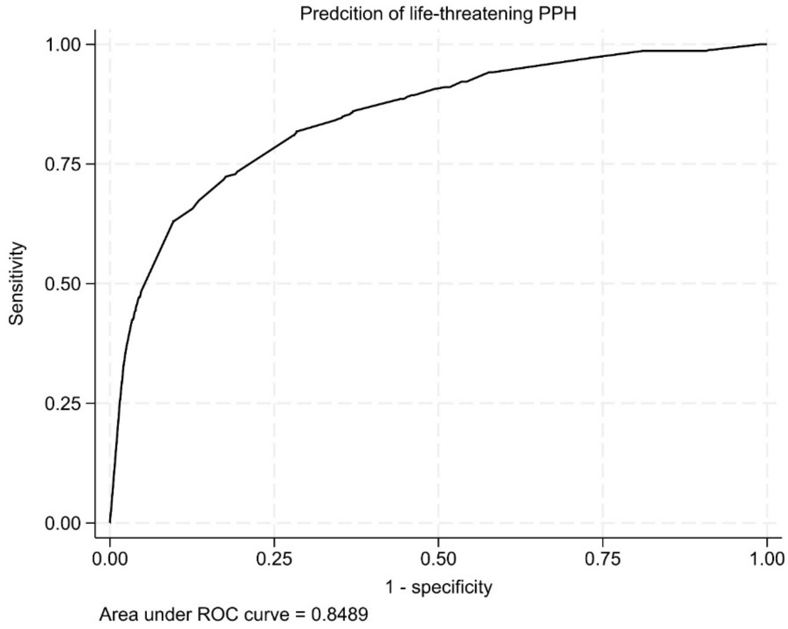

Classification of underlying risk categories

Low risk: Probability of life-threatening bleeding <2‰
High risk: Probability of life-threatening bleeding ≥2‰

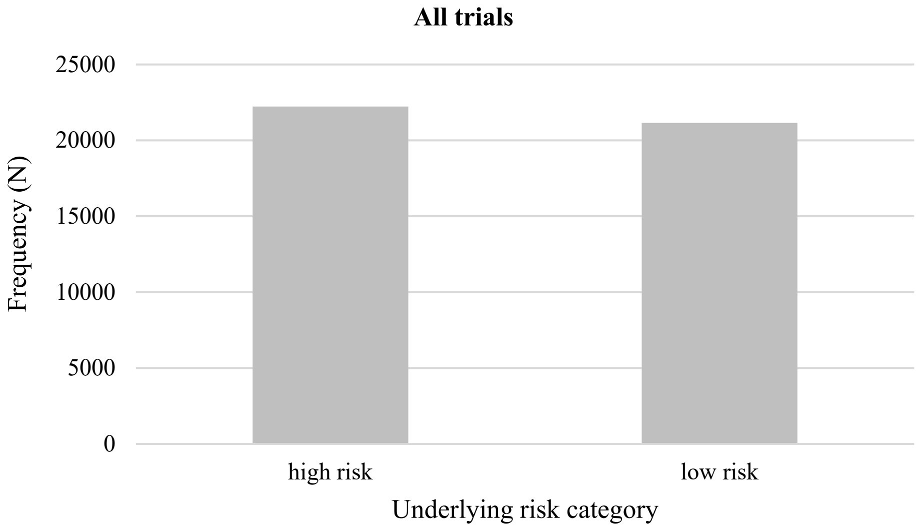

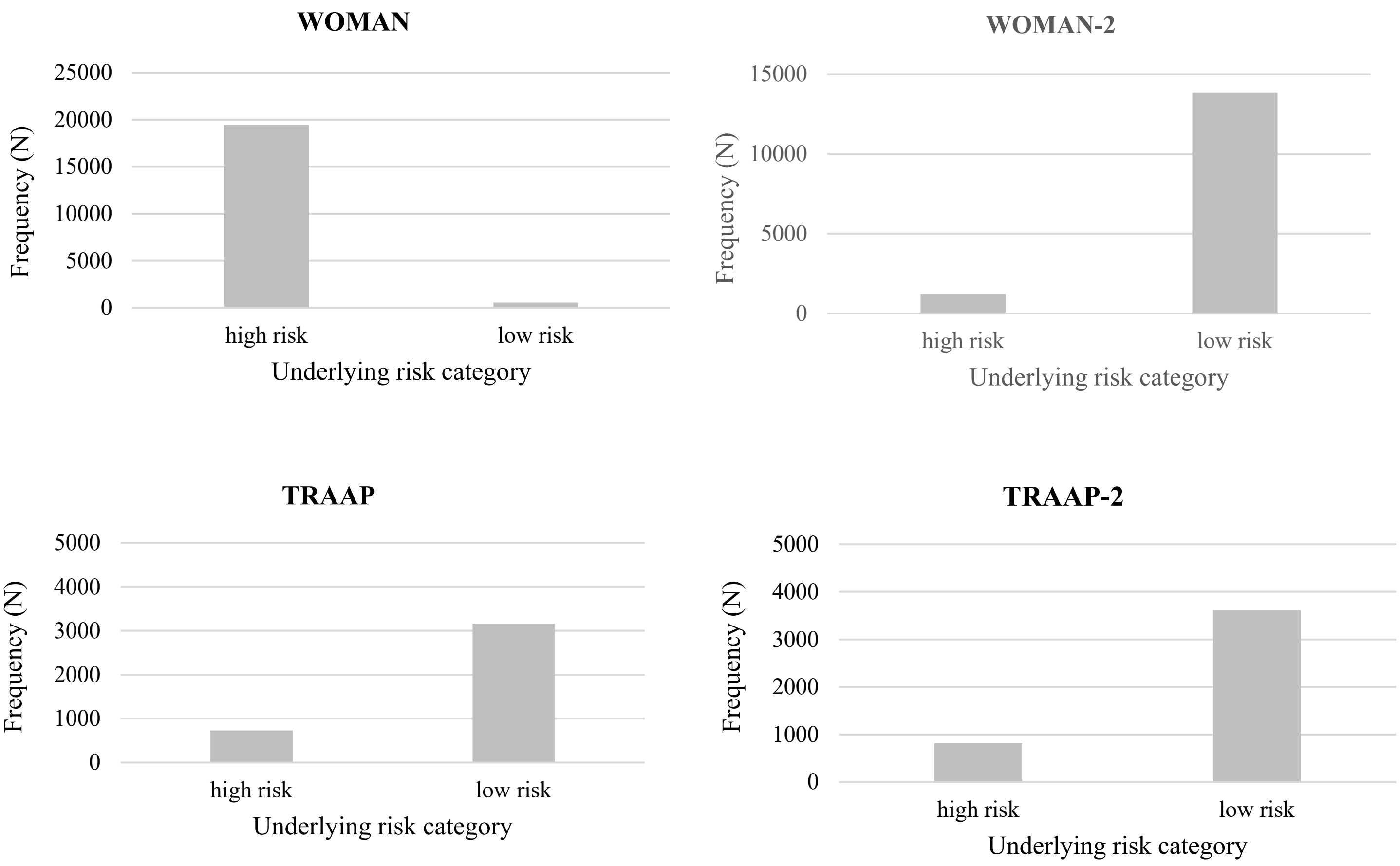

WOMAN
25000
20000
15000
10000
5000
0
Frequency (N)
high risk
low risk
Underlying risk category
WOMAN-2
15000
10000
5000
0
Frequency (N)
high risk
low risk
Underlying risk category
TRAAP
5000
4000
3000
2000
1000
0
Frequency (N)
high risk
low risk
Underlying risk category
TRAAP-2
5000
4000
3000
2000
1000
0
Frequency (N)
high risk
low risk
Underlying risk category

File 6: Effect of tranexamic acid on measures of maternal tiredness, anxiety and depression in the WOMAN, WOMAN-2, TRAAP and TRAAP-2 trials

	TXA n/N	Placebo n/N	OR (95% CI)
**WOMAN** – *measured as part of quality-of-life assessment using the EQ5D at hospital discharge or on day 42 if still in hospital*			
Severely anxious or depressed	30/9805	29/9728	1·03 (0·62-1·71)
**WOMAN-2** – *measured as part of the modified multi-dimensional fatigue symptom inventory administered during an interview with each woman at hospital discharge or on day 42 if still in hospital*			
Quite a bit or extremely depressed	181/7439	174/7378	1·03 (0·84-1·27)
Quite a bit or extremely worried or distressed	174/7439	137/7378	1·27 (1·01-1·59)
Quite a bit or extremely fatigued	268/7438	257/7377	1·04 (0·87-1·23)
**TRAAP** - *measured as part of maternal satisfaction and psychological assessment through a self-administered questionnaire on day 2 postpartum and EPDS by mail on day 60*			
Very or extremely tired	324/1526	347/1540	0·93 (0·78-1·10)
Very or extremely anxious	42/1526	57/1540	0·74 (0·49-1·10)
EPDS ≥12	156/1394	163/1381	0·94 (0·75-1·19)
**TRAAP-2** - *measured as part of maternal satisfaction and psychological assessment by self-administered questionnaire on day 2 postpartum and EPDS by mail on day 60*			
Very or extremely tired	385/1823	402/1811	0·94 (0·80-1·10)
Very or extremely anxious	86/1823	78/1811	1·10 (0·80-1·51)
EPDS ≥12	272/1398	238/1395	1·17 (0·97-1·42)

File 7: Effect of tranexamic acid on vomiting, dizziness and photopsia

	**TXA n/N**	**Placebo n/N**	**OR (95% CI)**
**Vomiting**			
WOMAN-2	66/7574	65/7484	1·00 (0·71-1·42)
TRAAP	73/1945	33/1946	2·26 (1·49-3·43)
TRAAP-2	940/2186	786/2166	1·32 (1·17-1·50)
TXA-MFMU	266/5069	273/4996	0·96 (0·81-1·14)
**Pooled**	**1345/16,774**	**1157/16,592**	**1·22 (1·11-1·34)**
**Heterogeneity p-value=0.0003**			

**Dizziness**			
WOMAN-2	413/7574	429/7483	0·95 (0·83-1.09)
TRAAP	40/1945	30/1946	1·34 (0·83-2·16)
TRAAP-2	93/2186	68/2166	1·37 (1·00-1·89)
TXA-MFMU	156/5069	186/4996	0·82 (0·66-1·02)
**Pooled**	**702/16,774**	**713/16,591**	**0·97 (0·87-1·08)**
**Heterogeneity p-value=0.03**			

**Photopsia**			
TRAAP	4/1945	6/1946	0·67 (0·19-2·36)
TRAAP-2	8/2186	2/2166	3·97 (0·84-18·74)
**Pooled**	**12/4131**	**8/4112**	**1·50 (0·61- 3·66)**
**Heterogeneity p-value=0.08**			

File 8: Summary of findings table and GRADE assessment (prepared using the MAGICapp platform - app.magicapp.org)

Outcome<br>Timeframe	Study results and measurements	Absolute effect estimates		Certainty of the evidence<br>(Quality of evidence)	Summary
		Placebo	Tranexamic acid		
Life-threatening bleeding[1]<br>Within 24 hour of giving birth	Odds ratio: 0·77<br>(CI 95% 0·63–0·93)<br>Based on data from 54,393 participants in 5 studies	9<br>per 1000	7<br>per 1000	High	Tranexamic acid decreases life-threatening bleeding
		Difference: 2 fewer per 1000<br>(CI 95% 3 fewer - 1 fewer)			
Thromboembolic events[2]<br>To end of follow-up for each trial	Odds ratio: 0·96<br>(CI 95% 0·65–1·41)<br>Based on data from 52,944 participants in 5 studies	2<br>per 1000	2<br>per 1000	Low<br>Due to serious inconsistency, Due to serious imprecision[3]	Too few women experienced a thromboembolic event to determine whether tranexamic acid made a difference
		Difference: 0 fewer per 1000<br>(CI 95% 1 fewer - 1 more)			
Clinically significant postpartum bleeding[4]<br>Within 24 hours after giving birth	Odds ratio: 0·97<br>(CI 95% 0·9–1·05)<br>Based on data from 23,370 participants in 3 studies	138<br>per 1000	134<br>per 1000	High	Tranexamic acid has little or no difference on clinically significant postpartum bleeding
		Difference: 4 fewer per 1000<br>(CI 95% 12 fewer - 6 more)			
Death<br>Within 24 hours after giving birth	Odds ratio: 0·76<br>(CI 95% 0·62–0·94)<br>Based on data from 54,404 participants in 5 studies	8<br>per 1000	6<br>per 1000	High	Tranexamic acid decreases death
		Difference: 2 fewer per 1000<br>(CI 95% 3 fewer - 0 fewer)			
Shock index ≥1·4<br>Within 24 hours after giving birth	Odds ratio: 0·97<br>(CI 95% 0·76–1·24)<br>Based on data from 22,997 participants in 3 studies	12<br>per 1000	12<br>per 1000	High	Tranexamic acid probably has little or no difference on shock index ≥1·4
		Difference: 0 fewer per 1000<br>(CI 95% 3 fewer - 3 more)			
Surgical intervention for bleeding<br>Within 24 hours after giving birth	Odds ratio: 0·79<br>(CI 95% 0·43–1·44)<br>Based on data from 23,377 participants in 3 studies	2<br>per 1000	2<br>per 1000	Moderate<br>Due to serious imprecision[5]	Too few women received a surgical intervention for bleeding to determine whether tranexamic acid made a difference
		Difference: 0 fewer per 1000<br>(CI 95% 1 fewer - 1 more)			
Peripartum haemoglobin change	Lower better<br>Based on data from 33,086 participants in 4 studies	g/LMean	g/LMean	Moderate<br>Due to evidence for statistical heterogeneity between trials (p=0·0002).[6]	Tranexamic acid probably lessens peripartum haemoglobin drop
		Difference: MD 0·64 lower<br>(CI 95% 0·39 lower - 0·89 lower)			

1. Death or surgical intervention for bleeding (laparotomy, embolisation, uterine compression sutures or arterial ligation) within 24 hours
2. ≥1 of the any of the following; myocardial infarction, stroke, deep vein thrombosis or pulmonary embolism as diagnosed by each trial up to the end of follow-up for each trial
3. Inconsistency: serious (no evidence for statistical heterogeneity, however point estimates vary widely and the direction of the effect is not consistent between the included studies); Imprecision: serious (wide confidence intervals).
4. Life-threatening bleeding or ≥1 of the following interventions for bleeding; additional uterotonics, non-trial TXA, perineal or vaginal packing, manual removal of placenta, uterine tamponade, bimanual compression, external aortic compression, non-pneumatic anti-shock garment, uterine compression sutures, arterial ligation, or arterial embolisation
5. Imprecision: serious (wide confidence intervals)
6. Inconsistency: serious (evidence for statistical heterogeneity [p=0·0002])
